# Exercise Therapy Interventions in Patients With Hip Osteoarthritis: Comparison of the Effects of DVD and Website-Based Interventions

**DOI:** 10.2196/rehab.8251

**Published:** 2018-05-07

**Authors:** Yuko Uesugi, Junichiro Koyanagi, Keishi Takagi, Ryota Yamaguchi, Shinya Hayashi, Takashi Nishii

**Affiliations:** ^1^ International Health Graduate School of Health Sciences Kobe University Kobe Japan; ^2^ Department of Orthopaedic Surgery Osaka General Medical Center Osaka Japan; ^3^ Department of Rehabilitation Osaka University Hospital Suita Japan; ^4^ Re concept Co, Ltd Kobe Japan; ^5^ Department of Orthopaedic Surgery Kobe University Graduate School of Medicine Kobe Japan

**Keywords:** hip osteoarthritis, exercise, DVD, website, self-efficacy

## Abstract

**Background:**

Prevalence of developmental hip dysplasia is high in Japan. Exercise therapy has been proven effective to treat certain aspects of hip osteoarthritis. Moreover, therapy provided via digital video discs (DVDs) and websites allows patients to exercise in the comfort of their own homes. However, no studies have evaluated the effectiveness of visual instructions in patients with hip disorders.

**Objective:**

This study aimed to compare the effectiveness of exercise therapy administered via DVD and that administered via a website.

**Methods:**

We developed a six-step progressive exercise therapy program for patients with hip osteoarthritis, which included three kinds each of open kinetic chain and closed kinetic chain exercises. Once the program was developed, exercise DVDs were produced. In addition to the six-step exercise program, our website was enabled to count the number of exercises performed by each patient and was accessible via the Internet at any time. Patients with hip osteoarthritis for whom surgery was not advised were enrolled by one university hospital in the Kansai area in Japan. Clinical symptoms and hip function were quantified using the Japanese Orthopedic Association Hip Disease Evaluation Questionnaire (JHEQ) and the Oxford Hip Score (OHS). Quality of life was measured using the SF-8 Health Survey, and self-efficacy for continued exercise was measured using the General Self-Efficacy Scale (GSES). Questionnaires were completed preintervention and after 6 months.

**Results:**

At 6-month follow-up, 10 DVD users (1 male, 9 female; mean age 51.3, SD 16.1 years) and 18 website users (2 male, 16 female; mean age 52.4, SD 10.4 years) were reachable. The change in each parameter could not be confirmed a significant improvement. However, most items tended to reflect overall improvement during the 6 months of intervention (*P*=.05-.94; paired *t* test). Regarding effect size, we considered a small effect to be greater than 0.2. Little effect was observed for JHEQ pain, SF-8 physical component summary (PCS), and SF-8 mental component summary in the DVD group, as well as OHS, SF-8 (PCS), and GSES in the website group.

**Conclusions:**

When comparing the effectiveness of exercise therapy between our DVD and website, we found that although both groups tended to improve in physical function, only the website group showed tendency of enhanced self-efficacy.

## Introduction

Many people needing care in daily life have low activity levels due to decreased musculoskeletal function [1]. Hip and knee osteoarthritis are the major causes of decreased physical function in this population. Prevalence of developmental dysplasia of the hip is high in Japan, and morbidity associated with hip osteoarthritis has been reported to be 1.0% to 4.3% [2]. It is estimated that these figures will continue to rise as the aging population grows.

Conservative methods to treat hip osteoarthritis include pharmaceutical treatment, exercise therapy, thermotherapy, and surgeries such as osteotomy and arthroplasty, depending on a patient’s general condition and progress in the alleviation of symptoms [3]. Among the conservative treatments, exercise therapy has the advantage of fewer adverse effects on internal organs, it can be practiced anywhere, and at little cost [4]. Moreover, exercise therapy in a patient’s home is effective for gait ability and activities of daily living owing to improvement in joint stability, muscle strength, and range of motion [5]. Previous studies have demonstrated that hip osteoarthritis patients can experience improvements in their pain and physical function through exercise therapy [6-8], and that this therapy is more effective when it is conducted in a patient’s home rather than at a hospital [9]. Therefore, it is important to provide an effective home-exercise program that can be easily understood, that can be performed at home by patients, and that is adaptable to their individual symptoms.

In recent years, visual instruction using digital video discs (DVDs) has been used to promote continued exercise in patients with knee osteoarthritis [10] and disuse syndrome [11]. Exercise therapy via DVDs and websites allows patients to exercise while watching video exercise demonstrations in the convenience of their own homes. However, DVD exercise programs can only be utilized with the delivery of a copied DVD to the patients. On the other hand, websites allow patients wider access to up-to-date exercise programs via the Internet and provide additional information via interactive communication, despite concerns regarding affinity to the Internet in older patients. Moreover, to our knowledge there are no studies that have evaluated the effectiveness of exercise videos in patients with hip disorder.

We developed a video exercise program and provided it through both a DVD and a website. Therefore, the purpose of this study was to compare the effectiveness of these modes of exercise therapy.

## Methods

### Developing the Exercise Program and Intervention Tool

As an intervention tool, we developed a six-step progressive exercise therapy program for patients with hip osteoarthritis. The program is a modification of an exercise program conceived by Conn et al [12] and Imada et al [13]. On developing the exercise program, the contents were tested by a team of three orthopedic surgeons specializing in lower extremity joints, two physical therapists providing rehabilitation exercise training for hip osteoarthritis patients, and one orthopedic research nurse. The program was finalized by confirming whether patients who were undergoing treatment for hip osteoarthritis were able to do the exercises.

**Figure 1:**
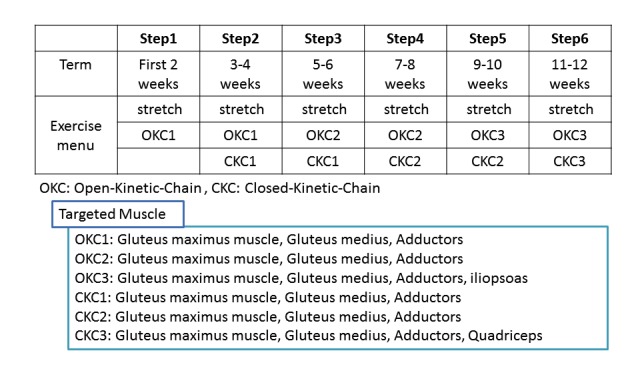
Model exercise program.

The six steps in the program consisted of three levels of both open kinetic chain (OKC) and closed kinetic chain (CKC) exercises. An OKC exercise refers to an exercise done while the limbs are free, and a CKC exercise is performed while limbs are on the floor. Both OKC and CKC exercises are considered to be most effective when done in combination [14]. The OKC exercises included hip contraction, hip abduction, hip external rotation, and side-lying hip abduction. The CKC exercises, performed with the patient standing, included hip contraction with hands placed on a table, adductor contraction using a ball, and knee flexion. The exercise menu was designed so that patients could reach final step (step 6) in a minimum of 3 months by advancing to new steps for both OKC and CKC exercises every 2 weeks, with 30 to 40 minutes of exercise a day (Figure 1).

Once the program was developed, a professional company produced the exercise DVD, which included easily comprehendible pictures and videos accompanied by relaxing music. The DVD instructed the user about the number of times each exercise should be performed to promote exercise continuity. Some exercises for adductor muscles required a ball, which was provided to the patients along with the DVD. We also constructed a website including the same exercise program as that on the DVD. In addition, this platform had the facility to count the repetitions of exercises performed by the patients, and was accessible to the patients via the Internet at any time. Moreover, the program allowed patients to record their pain levels by using the Numerical Rating Scale (NRS): ranging from zero for no pain to 10 for severe pain. We also developed a platform for counting exercise repetitions and for recording any comments. In addition, website group patients were provided with an exercise ball along with information about the website.

For both the DVD and website programs, we asked the patients to begin their regimens at an appropriate level. We recommended that participants only advance one step on either set every 2 weeks (Figure 1). If the exercise menu was not completed due to pain, we recommended that the patients discontinue the exercise for a couple of days and resume exercising after pain relief, with extension of the step for more than 2 weeks. After reaching the final step (step 6), we suggested that the patients continue the exercise at the step 6 level.

### Participants and Measurements

Patients with hip osteoarthritis were recruited into the study when surgery was not advised because of their levels of pain, activity impediments, and results of X-rays, or patients did not wish to receive surgery in the orthopedic outpatient facility of one university hospital in the Kansai area in Japan. From June 2011 to April 2012, we asked 24 patients; 17 patients who could use a DVD player were enrolled into the DVD study. From July 2014 to April 2015, we asked 40 patients, and 29 patients who had accessibility to the Internet were enrolled into the website study. The intervention times of the two groups were different because we confirmed the effect of the DVD first and then developed the website system in expectation of widespread use of exercise videos among patients.

Clinical symptoms and hip function were quantified using the Japanese Orthopedic Association Hip Disease Evaluation Questionnaire (JHEQ) [15] and the Oxford Hip Score (OHS) [16]. General quality of life (QOL) was measured by the SF-8 Health Survey [17] and self-directed continued exercise was measured using the General Self-Efficacy Scale (GSES) [18]. The exercise count for Web intervention users was recorded on the website.

Questionnaires were completed in an outpatient orthopedic setting before the intervention and after 6 months on the program. The questionnaires were provided in paper form for both groups of participants. We mailed the questionnaire to the homes of those patients who did not consult a doctor at the appropriate time.

### Measurement

The JHEQ, a useful, statistically reliable, and valid tool to evaluate patients with hip arthritis in Japan, is comprised of three categories—pain, movement, and mental. Each category consists of seven items, for a total of 21 items, which are used as evaluation criteria for hip joint function. Scores can range from zero to 28 points, with higher scores reflecting fewer symptoms and better functioning [15].

The OHS, a health-related QOL scale for hip arthritis patients, consists of 12 items, with higher scores reflecting better functioning and less pain. The scores can range from zero to 48 [16,19,20]. The reliability and validity of the OHS have been confirmed by a prospective study [21].

The SF-8 Health Survey is a generic, eight-item assessment that generates a health profile consisting of a physical component summary (PCS) and a mental component summary (MCS). The average value is 50 points, with higher scores indicating better functioning [22].

The GSES is a 16-item questionnaire measuring self-efficacy. The total score ranges from zero to 16 points, with higher scores indicating higher self-efficacy. Self-efficacy describes the belief that a person is capable of conducting their own actions independently. A number of literature reviews have examined the relationship between patient education and self-efficacy, suggesting that patient education increases self-efficacy and improves patients’ management skills [18,23,24]. At the end of this questionnaire, we provided recording space for patients to comment on their intervention.

### Intervention

The exercise menu was designed so that patients could reach the final step in a minimum of 3 months; however, patients were advised to discontinue the exercise for a couple of days when they felt remarkable pain, and continue the exercise program past 3 months.

We recommended that the exercises be carried out daily by providing the patients with a model exercise schedule. However, we also considered the pain and fatigue of the patient. We asked participants to schedule their exercise according to their physical condition and to take a break when they experienced pain. After reaching final step 6, the patients were asked to continue exercising at the step 6 level.

We also confirmed the difference between QOL and self-efficacy scores between preintervention and after 6 months for both the DVD and website groups considering effective period. Similarly, another study reported the effect of exercise intervention after 6 months [25].

### Ethical Considerations

Patients were informed that participation was voluntary, that they would not be treated unfavorably if they declined, that consent could be retracted at any time during the study, and that the research data would be coded to ensure confidentiality and privacy. We assigned an ID and password to each patient to protect their information on the website. The study was approved by the ethics committee of Kobe University Graduate School of Health Sciences. All participants provided written informed consent.

### Statistical Analysis

We compared the difference in mean data for both the DVD and website groups between preintervention and postintervention (paired *t* test). We also compared the difference in effect between the two groups. The effect size was calculated by dividing the difference in mean data by standard deviation.

## Results

A total of 17 eligible patients were enrolled in the DVD group, and 29 were enrolled in the website group. At the 6-month follow-up, seven patients in the DVD group (one male and six female; mean age 44.9, SD 13.9 years) did not respond to the mailed questionnaires. In the website group, two patients dropped out due to intolerable pain during exercise, and nine patients did not respond to the mailed questionnaires. Of the 11 patients who dropped out, one was male and 10 were female (mean age 38.2, SD 14.1 years). Therefore, 10 patients from the DVD group and 18 patients from the website group were included in our final analysis. Table 1 presents the characteristics of the 10 patients from the DVD group (1 male, 9 female; mean age 51.3, SD 16.1 years) and the 18 patients from the website group (2 male, 16 female; mean age 52.4, SD 10.4 years). Nonrespondents in the website group were younger than the respondents (*P*=.02).

**Table 1:** Characteristics of respondents and nonrespondents.

Characteristics				Respondents (n=28)		Nonrespondents (n=18)		*P* value^a^
**DVD group, n**				10		7		N/A^b^
	**Age (years)**							
		Mean (SD)	51.3 (16.1)		44.9 (13.9)		.73	
		Range	29-77		26-66		N/A	
	**Sex, n (%)**							
		Male	1 (10.0)		1 (14.3)		N/A	
		Female	9 (90.0)		6 (85.7)		N/A	
	BMI (kg/m^2^), mean (SD)			22.4 (3.1)		22.1 (3.5)		.96
**Website group, n**				18		11		N/A
	**Age (years)**							
		Mean (SD)	52.4 (10.4)		38.2 (14.1)		.02	
		Range	25-69		20-57		N/A	
	**Sex, n (%)**							
		Male	2 (11.1)		1 (9.1)		N/A	
		Female	16 (88.9)		10 (90.9)		N/A	
	BMI (kg/m^2^), mean (SD)			22.0 (3.0)		24.0 (10.0)		.70

^a^Mann-Whitney *U* test.

^b^N/A: not applicable.

**Table 2:** Scores of Japanese Orthopedic Association Hip Disease Evaluation Questionnaire (JHEQ), Oxford Hip Score (OHS), SF-8 Health Survey, and General Self-Efficacy Scale (GSES) for preintervention (pre) and after 6 months (post) in the digital video disc (DVD) and website groups.

Intervention		DVD (n=10)					Website (n=18)					
		Pre^a^, mean (SD)	Post^b^, mean (SD)	Diff^c^	ES^d^	*t*_9_		Pre, mean (SD)	Post, mean (SD)	Diff	ES	*t*_17_
**JHEQ**												
	Pain	13.5 (5.8)	15.6 (7.1)	2.1	–0.4	0.35		11.4 (6.7)	12.3 (7.0)	0.9	–0.1	0.52
	Movement	12.9 (9.5)	13.4 (8.9)	0.5	–0.1	0.69		11.7 (8.3)	11.6 (9.0)	–0.1	0.0	0.94
	Mental	17.6 (7.8)	18.6 (7.5)	1.0	–0.1	0.58		12.3 (8.0)	13.3 (7.6)	1.0	–0.1	0.46
OHS		38.1 (5.8)	37.5 (6.6)	–0.6	0.1	0.74		33.9 (8.7)	36.7 (7.5)	2.8	–0.3	0.05
**SF-8**												
	PCS	44.1 (6.2)	45.6 (7.1)	1.5	–0.2	0.70		43.2 (7.7)	44.8 (6.0)	1.6	–0.2	0.32
	MCS	52.3 (7.0)	54.1 (2.2)	1.8	–0.3	0.45		49.7 (5.0)	49.9 (7.2)	0.2	0.0	0.89
GSES		9.2 (4.7)	9.4 (4.8)	0.2	0.0	0.62		7.8 (4.1)	8.7 (4.6)	0.9	–0.2	0.09

^a^Pre: preintervention.

^b^Post: 6 months after intervention.

^c^Diff: difference.

^d^ES: effect size.

There were four bilateral osteoarthritis patients in the DVD group (40%) and three in the website group (17%). The change in each parameter during the 6 months (preintervention to postintervention) were as follows: JHEQ pain in the DVD group was 2.1 and in the website group was 0.9, JHEQ-movement was 0.5 in the DVD group and –0.1 in the website group, JHEQ mental was 1.0 in both the DVD and website groups, OHS was –0.6 in the DVD group and 2.8 in the website group, SF-8 (PCS) was 1.5 in the DVD group and 1.6 in the website group, SF-8 (MCS) was 1.8 in the DVD group and 0.2 in the website group, and GSES was 0.2 in the DVD group and 0.9 in the website group (Table 2). Although a significant improvement could be not confirmed, most items tended to reflect overall improvement during the 6 months of intervention (*P*=.05-.94; paired *t* test).

Regarding effect size, we considered a small effect to be greater than 0.2 [26]. Little effect was observed for JHEQ pain, SF-8 (PCS), and SF-8 (MCS) in the DVD group, as well as OHS, SF-8 (PCS), and GSES in the website group.

In the website group, seven patients counted the number of exercises. The range of counts was 6 to 47 (mean 24.6, SD 19.8) 6 months after intervention. In one day, the mean number of exercises was 4.6 (SD 3.6). Five patients recorded their pain by NRS on the website but continued to exercise. We received comments from nine patients in the website group by questionnaire; of these, two patients mentioned difficulty in continuing (eg, they had hoped to continue exercising at first, but were unable to do so because they were tired by their work).

## Discussion

### Principal Results

In both the DVD and the website groups, a majority of clinical and physical scores tended to improve; however, the difference in the effectiveness of the physical therapy between the two modes was not significant. However, effect size was greater than 0.2, indicating a small effect [26]. These findings suggest that the effectiveness of exercise therapy via both DVD and website is similar. Results of JHEQ pain and movement scores shows a tendency of relief of hip joint pain and expansion of movements related to hip joint function through the exercises provided. Tendency of improvement in SF-8 PCS scores on the general QOL scale suggests that not only can better hip joint function be achieved, but also function of movements can be improved, through exercises such as those outlined in this program. These improvements are reflected in JHEQ mental and SF-8 (MCS) scores in this study.

After 6 months, the effect size was greater than 0.2 as reflected by JHEQ pain, SF-8 (PCS), and SF-8 (MCS) scores in the DVD group, and OHS, SF-8 (PCS), and GSES scores in the website group. Tendency of improved physical function is reflected in the SF-8 (PCS) scores seen in both groups. This may be the result of both groups performing the same exercises. Improvement in preintervention to postintervention JHEQ pain scores in the DVD group was 2.1, which was higher than that of the website group (0.9). However, the effect size of OHS was higher in the website group than that in the DVD group. In the website group, the ratio of unilateral patients was high, which suggests the possibility of improvement in function toward that of normal. In the website group, a tendency of improved GSES scores was seen. To enhance self-efficacy, it is preferable to guide the patients to help them obtain a sense of achievement, which is important for behavior modification [18].

In the website group, seven participants recorded that their number of exercise repetitions (range 6-47 times; mean 24.6, SD 19.8). The mean number of exercises was 4.6 (SD 3.6) per one day. The number of exercises required for each step was different, according to patients. However, the trending improvement of their physical function suggests some patients continued exercise although they did not count. We have to confirm that they developed an exercise habit.

In a previous study investigating the effect of exercise intervention, the group receiving normal care and additional exercise therapy showed improvements after 3 months; however, after 12 months there was no significant difference [27]. Likewise, the study of a Web-based intervention showed significant improvements after 3 months, but these were not significant when compared to those of the control group after 12 months [28]. Moreover, it has been reported that adherence to continuing positive behavior is related to patients recognizing the benefits of exercise, and that the problem is the lack of a patients’ perception of exercise’s long-term effects [29].

Our findings suggest that some patients might quit exercise after 6 months because of the difficulty in continuing. However, patients who were recorded on the website commented that they could not continue although they had hoped to at first. At the beginning of the intervention, patients held a high level of interest, but their motivation decreased gradually. A previous study reported that it was difficult for patients to keep up an effective rate of continuing exercise without continued intervention [30]. It is suggested that more intervention to enhance their motivation was needed during 6 months.

In our study, the patients in the DVD group could only exercise by watching the video, whereas those in the website group had to count their exercise repetitions. This suggests that they felt a sense of achievement toward accomplishing their goals, which in turn, improved their self-efficacy scores. Thus, a website-based intervention might improve the motivation to exercise.

In this study, we provided two interventions—DVD and website—and both of these were effective. The mean ages of the DVD and website groups were not significantly different. This suggests that patients who have use of an intervention medium get effective exercise. Some elderly patients might not use the Internet and it is not convenient for them. Therefore, it is good for patients to be able to choose the medium that is the most convenient for them.

Although this study showed no difference in effectiveness between the DVD and website groups, the website platform may be more useful because instructors have the ability to modify both the interface and exercise program via the Internet at any time. Recently, there have been an increasing number of studies investigating tools that make use of websites. Moreover, the effects of Web interventions have been reported as resulting in greater learning and changes in behavior than have other modes of intervention [31]. However, patients using a website require individual information, support, and feedback; therefore, it is important to develop a website to allow better interaction between patients and to provide individualized feedback regarding patients’ progress and outcomes [32].

### Limitations

One limitation of this study was the low retention rate. The nonrespondent patients were younger than the respondent patients were. The younger patients tended to play many roles in society, such as working and parenting; therefore, they may have had difficulty continuing this study and responding to us. Another study limitation was that the sample size was small and the differences in the results were not statistically significant. The most probable cause of this divergence was stochastic variation. Finally, there was insufficient diversity in the patients because they were all enrolled from the same facility. Most patients were in their fifties, so the study sample was predominantly a middle-age group. Therefore, in the future, we will design a system and conduct a study for use with elderly hip osteoarthritis patients.

### Conclusions

We investigated the effects of exercise therapy interventions via DVD and a website. After 6 months, the effect size was greater than 0.2 as reflected by JHEQ pain, SF-8 (PCS), and SF-8 (MCS) scores in the DVD group, and OHS, SF-8 (PCS), and GSES scores in the website group. Although both groups we studied tended to report improved physical function, only the website group reported a tendency of enhanced self-efficacy—an important factor in behavior modification. Therefore, it is vitally important that we continue studying the use of website-based interventions for patients suffering from hip osteoarthritis.

## Abbreviations

CKC: closed kinetic chain
DVD: digital video disc
GSES: General Self-Efficacy Scale
JHEQ: Japanese Orthopedic Association Hip Disease Evaluation Questionnaire
MCS: mental component summary
NRS: Numerical Rating Scale
OHS: Oxford Hip Score
OKC: open kinetic chain
QOL: quality of life
PCS: physical component summary
